# Deep Sequencing of the Transcriptomes of Soybean Aphid and Associated Endosymbionts

**DOI:** 10.1371/journal.pone.0045161

**Published:** 2012-09-12

**Authors:** Sijun Liu, Nanasaheb P. Chougule, Diveena Vijayendran, Bryony C. Bonning

**Affiliations:** Department of Entomology, Iowa State University, Ames, Iowa, United States of America; Natural Resources Canada, Canada

## Abstract

**Background:**

The soybean aphid has significantly impacted soybean production in the U.S. Transcriptomic analyses were conducted for further insight into leads for potential novel management strategies.

**Methodology/Principal Findings:**

Transcriptomic data were generated from whole aphids and from 2,000 aphid guts using an Illumina GAII sequencer. The sequence data were assembled *de novo* using the Velvet assembler. In addition to providing a general overview, we demonstrate (i) the use of the Multiple-*k*/Multiple-*C* method for *de novo* assembly of short read sequences, followed by BLAST annotation of contigs for increased transcript identification: From 400,000 contigs analyzed, 16,257 non-redundant BLAST hits were identified; (ii) analysis of species distributions of top non-redundant hits: 80% of BLAST hits (minimum e-value of 1.0-E3) were to the pea aphid or other aphid species, representing about half of the pea aphid genes; (iii) comparison of relative depth of sequence coverage to relative transcript abundance for genes with high (membrane alanyl aminopeptidase N) or low transcript abundance; (iv) analysis of the *Buchnera* transcriptome: Transcripts from 57.6% of the genes from *Buchnera aphidicola* were identified; (v) identification of *Arsenophonus* and *Wolbachia* as potential secondary endosymbionts; (vi) alignment of full length sequences from RNA-seq data for the putative salivary gland protein C002, the silencing of which has potential for aphid management, and the putative *Bacillus thuringiensis* Cry toxin receptors, aminopeptidase N and alkaline phosphatase.

**Conclusions/Significance:**

This study provides the most comprehensive data set to date for soybean aphid gene expression: This work also illustrates the utility of short-read transcriptome sequencing and the Multiple-*k*/Multiple-*C* method followed by BLAST annotation for rapid identification of target genes for organisms for which reference genome sequences are not available, and extends the utility to include the transcriptomes of endosymbionts.

## Introduction

Aphids are among the most economically important pest insects of temperate agriculture [1]. In addition to the major economic losses resulting from aphid feeding, aphids also transmit plant viruses [2], [3]. More than 450 species within the Aphididae deleteriously impact horticultural and agricultural commodities, of which more than 100 are categorized as pests of significant economic importance [1]. Indeed, aphid damage is so pervasive that accurate estimates of total losses are difficult to obtain. The pea aphid, *Acyrthosiphon pisum*, has emerged as a model species for analysis of both fundamental and applied aspects of aphid biology [4], [5] and the pea aphid genome has been sequenced [6]. The genomic resources available for aphid species other than the pea aphid are currently limited [7].

In North America and parts of Canada, the soybean aphid, *Aphis glycines* Matsumura (Hemiptera: Aphididae), has been of particular concern since its detection in the region in 2000 [8]. The soybean aphid infests two disparate plant species, and undergoes sexual reproduction on the primary host species (European buckthorn, *Rhamnus cathartica* in North America), and asexual reproduction on the secondary host (soybean, *Glycines max*) [8]. Soybean aphid populations can double every 6 to 7 days [9], with adults producing more than 9 nymphs per day [10]. Management of this invasive pest, which relies primarily on the application of foliar insecticides, is estimated to have cost $1.6 billion over the last decade [11]. Genetic analysis of the soybean aphid suggested that genetic diversity is limited within North America [12]. However, although soybean aphid resistance genes (***R*** *esistance to* ***A*** *phis* ***g*** *lycines*; *Rag*) have been identified in soybean varieties [13], biotypes of aphids that overcome this resistance were identified even before commercial release of the resistant lines [14], [15]. The mechanisms underlying soybean aphid resistance to resistant soybean are unknown. A compounding problem is the potential of the soybean aphid to vector plant viruses, including Alfalfa mosaic virus, Soybean mosaic virus, Cucumber mosaic virus, and potentially Soybean dwarf virus [16]. Novel approaches for management of this pest are clearly warranted.

Aphids are closely associated with bacterial endosymbionts, specifically with *Buchnera aphidicola*, a primary, obligatory species which resides in specialized cells, bacteriocytes, within the aphid. The primary role of these obligatory endosymbionts is to provide essential amino acids that are not synthesized by the host aphid [17]. The development of genomic resources for other aphid species has facilitated a more complete understanding of the interaction between *Buchnera* and the host aphid [18], [19]. In addition, aphids harbor secondary or facultative endosymbionts such as *Hamiltonella, Rickettsia*, *Arsenophonus, Regiella, Serratia* and *Wolbachia*. These symbionts function in aphid defense against pathogens and parasitoid wasps, and may be involved in resistance to host plant defense resulting in formation of aphid “biotypes” [20], [21], [22]. Secondary endosymbionts may be lost, or gained via both vertical and horizontal transmission [23].

Given the economic importance of the soybean aphid, genomic sequence resources for this agricultural pest are essential for (i) increased understanding of the biology and physiology of this species, (ii) identification of potential targets in the gut for novel aphicidal technologies (as the gut is readily accessible to ingested control agents, it provides a primary focus for novel pest control strategies), and (iii) monitoring of *A. glycines* biotypes in North America. Silencing of *C002* [24], [25], and the potential use of Bt-derived toxins against aphids [26] are of particular interest. We employed next-generation sequencing technology (Illumina Genome Analyzer II) to increase the molecular resources available for the soybean aphid. In addition to demonstrating the use of the Multiple-*k*/Multiple-*C* method for *de novo* assembly of short read sequences following by BLAST annotation of contigs, we addressed (i) analysis of species distributions of top hits, (ii) gene ontology analysis and comparison of whole aphid (WA) and gut transcriptomes, (iii) comparison of the soybean aphid transcriptome with pea aphid gene sets, (iv) comparison of relative depth of sequence coverage to relative transcript abundance for genes with high or low transcript abundance, (v) analysis of the *Buchnera* transcriptome, (vi) identification of *Wolbachia* and Arsenophonus as potential secondary endosymbionts of the soybean aphid, (vii) alignment of full length sequences from RNA-seq data. Our dataset has more than doubled the number of unique genes reported for the soybean aphid [27], and provides valuable datasets for further analyses of the soybean aphid gut and endosymbiont transcriptomes.

## Results and Discussion

### De novo assembly of Illumina short read sequences

Analysis of RNA-seq short read sequences presents a challenge for organisms for which genomic sequence data are not available. For *de novo* assembly, the Velvet program was used to generate contiguous sequences (contigs) [28]. In order to acquire maximum information from the RNA-seq data, we used the Multiple-*k* (hash length *k-mer*) method [29] combined with the multiple *C* (coverage cutoff) to generate multiple sets of contigs. The contig sets were depleted using the CD-HIT program [30] to reduce redundancy, and the resulting contigs for each sample (WA or gut) were combined. The two sets of pooled samples were again depleted with CD-HIT, and the numbers of contigs in each set (gut and WA) reduced to about 16% of the original number of contigs.

The final number of contigs for the soybean aphid gut transcriptome was 141,532 (> = 100 nt: Table 1) with the longest contig being 11,376 nt in length, and the average length being 424 nt. Twenty-five % (35,000) of the contigs were equal or greater than 500 nt in length. The final number of contigs for the whole soybean aphid (WA) transcriptome was 253,603 with an average contig length of 312 nt. Around 15.5% (39,600) of the contigs were equal to or longer than 500 nt, with the longest being 6,350 nt. These final contig sets covered about 80% of the reads from the gut sample and 64% of the reads from the WA sequences.

**Table 1:** Summary of BLAST analysis and annotation of soybean aphid sequences.

Contig sets	Gut	Whole Aphid
Total contigs (> = 100 nt)	141,532	253,603
Total BLASTx hits	100,230 (70.8%)*	185,650 (73.2%)
Total BLASTn hits	28,071(19.8%)	44,788(17.6%)
No hits	13,231 (9.4%)	23,185(9.1%)
Non redundant top hits (BLASTx)	10,640	14,861
Non-redundant BLASTx top hits to each set	1,396	5,617
Non redundant top hits (BLASTn)	6,396	9,862
**No redundant EC (Enzyme code)**	**527**	**641**
Non-redundant EC numbers for each set	68	182
% of contigs with at least 1 GO term	18.23	21.04
Inter Pro (Protein signatures)	1,775	1,478
Non-redundant Inter Pro to each set	808	440

* % of total number of contigs

The contig set for the gut transcriptome with the highest N50 was created by using *k* = 31 and *C* = 6. BLASTx analysis of this set of contigs resulted in identification of 3,931 non-redundant top hits. In comparison, by combining multiple contig sets, 10,640 non-redundant hits were identified (Table 1). Thus, the use of multiple contig sets with varying parameters, allowed for identification of 63% more soybean aphid transcripts than use of the single “optimal” set. Two sets of contigs (soybean aphid gut, whole aphid) have been deposited to AphidBase (http://www.aphidbase.com/aphidbase/).

### BLAST annotation of soybean aphid contigs

The final contig sets for the gut and WA transcriptomes were annotated with BLASTx against the NCBI nr database. Contigs without hits from BLASTx analysis were then annotated with BLASTn for detection of additional gene sequences (Table 1). The majority of the contigs (90.7% for the gut, and 90.8% for the WA) had hits with either BLASTx or BLASTn. Of these, hits were identified for 70.8% of the gut and 73.2% of the WA contigs by BLASTx. Analysis of contigs without BLASTx hits showed that 19.8% of the gut and 17.6% of the WA contigs hit nucleotide sequences on analysis with BLASTn (Table. 1). The majority of the contigs that did not align with either protein or nucleotide sequences on BLAST analysis were short contigs: 75% of the contigs that had no hits were less than 200 nt in length.

After removing redundant hits, we identified 10,640 and 14,861 non-redundant proteins from the gut and WA transcripts, respectively. Among the non-redundant hits, 9,244 (56.9%) were identified from both the gut and WA transcriptomes, while 1,396 (8.6%) were unique to the gut transcriptome, and 5,617 (34.6%) were unique to the WA transcriptome (Table 1). In total 16,257 unique protein hits were identified by BLASTx. Notably, as a result of both the sequencing and assembly methods employed, the number of non-redundant genes identified using the short read transcriptome sequencing approach was more than double the number reported using Roche-454 and Illumina GA II 51 bp – paired end reads [27].

Examination of the species distributions of the non-redundant top hits from both BLASTx and BLASTn showed that 83.0/91.1% (BLASTx/BLASTn) of the hits from the gut transcriptome and 75.7/91.4% of the hits from the WA transcriptome aligned to genes of the pea aphid and other aphid species (Table 2). A total of 4.1/1.8% of the WA top hits were genes of the endosymbiotic bacterium *Buchnera*.

**Table 2:** Species distribution of non-redundant top BLASTx hits for soybean aphid transcripts.

	Gut		Whole aphid	
Species	No. of non-redundant top hits	%	No. of non-redundant top hits	%
**Hemiptera**				
*Acyrthosiphon pisum*	8,730	82.05	11,141	74.97
Other aphid species	104	0.98	110	0.74
**Coleoptera**				
*Tribolium castaneum*	120	1.13	223	1.50
**Diptera**				
Mosquitos	131	1.23	233	1.57
*Drosophila spp.*	151	1.42	262	1.76
**Anoplura**				
*Pediculus humanus*	95	0.89	163	1.10
**Hymenoptera**				
*Harpegnathos saltator*	56	0.53	85	0.57
*Camponotus floridanus*	49	0.46	84	0.57
*Nasonia vitripennis*	64	0.60	84	0.57
*Solenopsis invicta*	41	0.39	67	0.45
*Acromyrmex echinatior*	31	0.29	50	0.34
*Apis mellifera*	43	0.40	64	0.43
*Bombus impatiens*	30	0.28	49	0.33
*Bombus terrestris*	35	0.33	36	0.24
**Lepidoptera**				
*Danaus plexippus*	34	0.32	65	0.44
***Endosymbiotic bacteria***				
*Buchnera aphidicola*	20	0.19	602	4.05
*Wolbachia*	0	0	3	0.02
Others	918	8.63	1,530	10.30
**Total non-redundant hits**	**106,40**	**100**	**14,861**	**100**

### Comparison of soybean aphid and pea aphid genes

**Figure 1:**
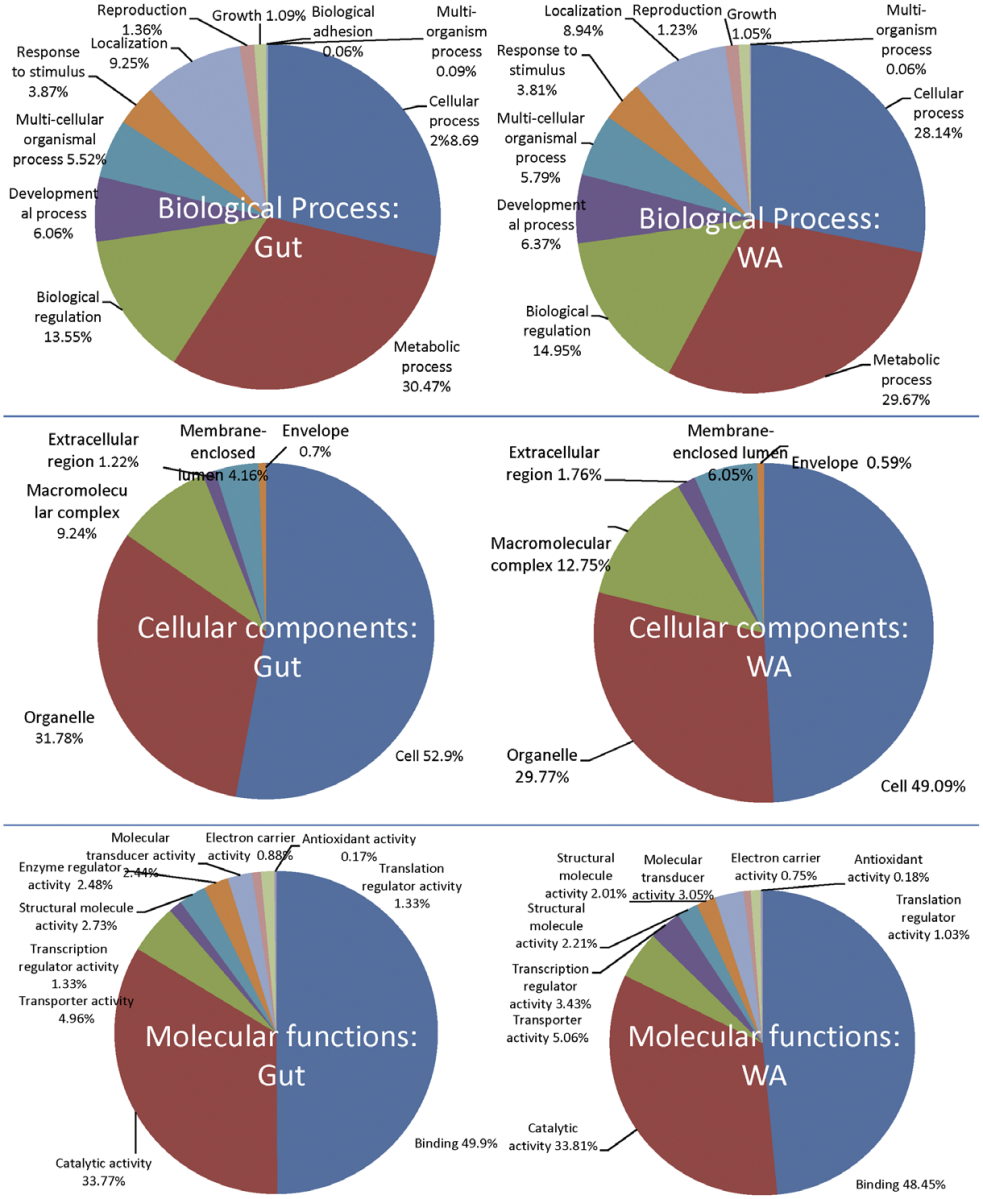
Distribution of soybean aphid sequences by gene ontology. (GO: level 2; filtered by sequence number cutoff  = 5) for biological process, cellular components, and molecular functions. Data are shown for both the gut (at left) and whole aphid (at right) transcriptomes.

To conduct a functional analysis of the soybean aphid genes, we tested various databases for gene annotation, including the NCBI database, Flybase (FlyBase http://flybase.org/) [31] and Swiss-Prot. Mapping of the soybean aphid transcriptome contigs against the protein sequences in the Swiss-Prot protein database by BLAST2GO resulted in identification of the most GO terms. Overall, only 18.2% of the gut contigs and 21.0% of the WA contigs were assigned at least one GO term (Table 1). Analysis of GO distributions showed similar GO distribution patterns between the gut and WA sequences (Figure 1). GO-enzyme code mapping assigned 709 non-redundant EC codes. Of those, 68 (9.6%) of the enzymes were unique to the gut and 182 (25.7%), were only identified in the WA samples (Table 1).

BLAST analysis of the soybean aphid transcriptome resulted in identification of more than 16,000 potential transcripts from the soybean aphid, which included transcripts from both the aphid and associated endosymbionts. Although some 35,000 genes are predicted from the pea aphid genome [6], it is unknown how many of the predicted genes are transcribed. Identification of sequences in the soybean aphid transcriptome homologous to predicted pea aphid genes supports transcription of these hypothetical genes. The pea aphid genome is remarkable in having a high level of gene duplication and expansion of some gene families. Such gene duplication and gene expansion events could impact the quality of de novo transcript assembly. The impact of this on the transcript assembly reported herein will become apparent once the soybean aphid genome sequence is available.

We used pea aphid genes as reference genes to search the soybean aphid transcriptome for genes homologous to those predicted or identified from the pea aphid genome. Seventeen groups of annotated pea aphid genes were selected for analysis (Table 3), which have a total of 1,430 genes with assigned IDs. Examination of the genes revealed that 1,145 (80.1%) of the 1,430 pea aphid genes had putative homologs in the soybean aphid sequences. Genes functioning in amino acid transport and sugar transport had the highest sequence identity between the two aphid species, with 95.7% of the amino acid transporter genes and 94.7% of the sugar transporter genes identified in the soybean aphid transcriptomes. In contrast, only 52.7% of the cathepsin genes (an important protease superfamily) of the pea aphid were identified in the soybean aphid transcriptomes. This result may indicate either that the putative cathepsin genes are not all expressed, possibly because of the high level of gene duplication in aphids and loss of function in some cases, or may reflect the tight regulation of expression of tissue specific cathepsin genes [32].

**Table 3:** Putative soybean aphid homologs to pea aphid gene sets.

			Number of homologs in soybean aphid								
Gene groups		No. genes in Pea aphid	Gut		(% of PA)	Whole aphid	(% of PA)	Total		(% of PA)	References
Amino acid biosynthesis		82	69	(84.15)		70	(85.37)	71	(86.57)		[18]
Amino acid degradation		119	99	(83.19)		99	(83.19)	101	(84.87)		[18]
Amino acid transporters		47	35	(74.47)		44	(93.62)	45	(95.74)		[18]
Cathepsins		74	35	(47.3)		39	(52.7)	39	(52.7)		NCBI database
Chitinase-like proteins		9	7	(77.78)		8	(88.89)	8	(88.89)		[61]
Chromatin remodeling proteins		145	71	(48.97)		94	(64.83)	94	(64.83)		[62]
Clock genes		14	11	(78.57)		11	(78.57)	12	(85.71)		[63]
Developmental		315	187	(59.37)		268	(85.08)	270	(85.71)		[64]
Homeobox		55	15	(27.27)		43	(78.18)	44	(80)		[64]
Immune and stress		98	54	(55.1)		68	(69.39)	68	(69.39)		[65]
Ion channels		85	30	(35.29)		66	(77.65)	66	(77.65)		[66]
Meiosis and cell cycle		80	43	(53.75)		64	(80)	65	(81.25)		[67]
Nuclear receptors		14	8	(57.14)		11	(78.57)	11	(78.57)		[68]
Purine metabolism and urea cycle		52	34	(65.38)		44	(84.62)	44	(84.62)		[19]
Sugar transporters		75	51	(68)		69	(92)	71	(94.67)		[69]
Transcytosis		146	94	(64.38)		115	(78.77)	117	(80.14)		[70]
Wing development		20	6	(30)		19	(95)	19	(95)		[71]
**Total**		1430	849	(59.37)		1132	(79.16)	1145	(80.07)		

Specific analysis to identify transcripts of digestive enzymes in the soybean aphid gut transcriptome resulted in identification of transcripts for alpha-amylase (8 BLASTx hits), aminopeptidase (17), carboxypeptidase-like (13), cysteine protease (2), and oligopeptidase (1); Transcripts potentially involved in detoxification included those for cytochrome P450-like (22 BLASTx hits), catalase (1), ferritin (3), glutathione S-transferase (4), peroxidase (5), peroxiredoxin (3), superoxide dismutase (1), and glutathione synthetase (1).

In the absence of the soybean aphid genome sequence or replication of the transcriptome sequencing, it is not possible to quantify variation in gene expression between the gut and the whole aphid. However, a comparison of the numbers of annotated genes between the two transcriptome data sets provides indicators of differential expression of gene types. For example, 55 homeobox genes have been annotated for the pea aphid. Of those, only 15 (27.3%) were identified in the soybean aphid gut transcriptome, but 43 (78.2%) were found in the WA transcriptome. In addition and as expected for genes related to wing development, only 6 out of 20 genes identified in the pea aphid were identified in the gut sequences, whereas 19 of the genes were identified in the WA sequences. Similar results were seen for genes involved in development and for genes encoding ion channels (Table 3).

Interestingly, 36 sequences from the gut transcriptome, and 46 from the whole aphid transcriptome had high homology to sequences from barley, *Hordeum vulgare* on BLASTx analysis. Further analysis with BLASTn indicated that these sequences are indeed aphid-derived (Table S1).

### Examination of relative transcript abundance

RNA-seq can be used for measuring relative transcript levels [33]. Expression levels are determined by comparing the relative depth of sequence coverage to assembled contigs, followed by qRT-PCR to confirm the relative abundance of selected transcripts. Because no genomic and only limited gene sequence information is available for the soybean aphid, it was not appropriate to determine the relative gene expression level by the RPKM value (i.e. reads per kilobase of exon model per million mapped reads). To assess the relative abundance of transcripts in the gut and WA samples, we mapped the 75 nt Illumina reads to the assembled contigs from the gut and WA using the MAQ program. The 10 contigs from the gut and WA samples with the highest depth of reads (and implied highest transcript abundance) are listed in Table 4. There is no overlap between the 10 most abundant transcripts from the soybean aphid WA and gut transcriptomes (Table 4). The RNA-Seq - predicted most abundant transcripts in the gut were for genes involved in amino acid and sugar metabolism. Of the five most highly expressed transcripts from the gut, three encode membrane alanyl aminopeptidase N (APN). This result is consistent with examination of APN expression in the pea aphid gut, which showed that APN is the most abundant protein comprising an estimated 16% of the total gut protein [34]. In that study, only one APN protein was isolated, while our gut transcriptome analysis showed that at least three APN-like genes were highly transcribed.

**Table 4:** Ten most abundant transcripts in the gut and whole aphid (WA) transcriptomes based on depth of reads assembled into contigs.

Gut	Putative Genes	Reads assembled	Top hit ID	Species	E-value	Identity (%)
1	Membrane alanyl aminopeptidase N	65316	XP_001944286.1	*A. pisum*	7.00E-52	75
2	Sugar transporter 1	42057	ACT10281.1	*Sitobion avenae*	2.00E-134	92
3	Membrane alanyl aminopeptidase N	31032	NP_001119606.1	*A. pisum*	1.00E-104	70
4	Membrane alanyl aminopeptidase N	29218	XP_001948350.1	*A. pisum*	1.00E-174	75
5	putative cathepsin B-S	22217	AAU84936.1	*Toxoptera citricida*	1.00E-87	86
6	Putative ADP/ATP translocase	21521	XP_001948359.1	*A. pisum*	2.00E-164	98
7	glutamine synthetase 2	20208	NP_001153848.1	*A. pisum*	3.00E-53	97
8	Alkaline phosphatase homologues	19840	XP_001943535.1	*A. pisum*	2.00E-54	80
9	Ac1147-like protein	16889	ABG74714.1	*Diaphorina citri*	4.00E-141	96
10	cathepsin B-16A	14523	NP_001119617.1	*A. pisum*	4.00E-122	86
**WA**						
1	similar to cytochrome P450	6741	XP_001944205.1	*A. pisum*	4.00E-162	92
2	similar to AGAP010734-PA, partial	6057	XP_001949485.1	*A. pisum*	0	97
3	similar to Collagen	5934	XP_001944753.1	*A. pisum*	0	76
4	DnaJ-like protein	5554	NP_001119620.1	*A. pisum*	8.00E-148	95
5	similar to cement precursor protein 3B	5029	XP_001945547.1	*A. pisum*	1.00E-113	67
6	ATP synthase-beta	5028	NP_001119645.2	*A. pisum*	4.00E-95	99
7	similar to paramyosin, long form	5023	XP_001948420.1	*A. pisum*	0	95
8	similar to Mitochondrial phosphate carrier protein	5009	XP_001945337.1	*A. pisum*	2.00E-166	95
9	similar to tyrosine hydroxylase	4616	XP_001944964.1	*A. pisum*	0	83
10	similar to proteophosphoglycan ppg1	4316	XP_001948991.1	*A. pisum*	4.00E-115	74

The depth of reads per putative gene for the 10 mostly highly expressed genes in the gut sample varied 4.5 fold (14,523 to 65,316 reads assembled). In contrast, the numbers of reads per gene for the most highly expressed transcripts in the WA sample, varied only 1.4 fold (4,316 to 6,741). Considering that the whole aphid RNA samples included all tissues and aphids in different developmental stages, it is not surprising to see reduced depth of coverage compared to the tissue specific transcriptome.

To confirm that the number of short reads assembled for a particular cDNA (mRNA-Seq) provided an indication of relative transcript abundance, we conducted qRT-PCR on total aphid RNA for four genes with high or low transcript abundance: two aminopeptidases, which were among the most abundant transcripts in the gut transcriptome, and two randomly selected genes of unknown function, with low transcript abundance (Table 5). While the numbers of reads assembled and relative abundance as determined by qRT-PCR are not well correlated, the fold-change when comparing treatments or tissues, correlates strongly with qRT-PCR results for a given gene (r = 0.966, n = 714 genes; Illumina RNA Analysis data sheet).

**Table 5:** qRT-PCR analysis of relative transcript abundance compared to mRNA-Seq data*.

Transcripts	Relative abundance (qRT-PCR)	Reads assembled	Length of contigs	Average sequence coverage (depth^a^)
FT1	1.00	2,775	934	222.80
FT2	8.04	1,725	520	248.79
APN3	25.46	31,032	2,725	854.09
APN4	24.84	29,218	1,471	1,489.70

* Selected cDNAs with abundant (APN3, APN4) and few (FT1, FT2) transcripts in the soybean aphid gut transcriptome were amplified by qRT-PCR with GAPDH as internal control. The relative abundance of each transcript as determined by qRT-PCR is shown alongside the numbers of short read sequences assembled for each cDNA.

a depth (coverage per nt)  =  number of reads x 75 nt/contig length.

### Buchnera aphidicola transcriptome

The genomes of symbiotic bacteria in the genus *Buchnera* are highly reduced. The *Buchnera* genome size is 14% that of the E. *coli* genome [35], [36] and is predicted to encode only 583 genes (*Buchnera sp*. APS) [37], which is only 3-fold the core sequence of a minimal bacterial gene set [38]. Because of the importance of these endosymbionts to aphid survival, we also examined the transcript profiles of the soybean aphid endosymbionts.

One of the WA RNA samples underwent a single polyA RNA purification step, rather than the two recommended by the Illumina RNA sample preparation protocol. As a result of this change in the protocol, approximately 30% of the RNA reads generated lacked a 3′ polyA tail.

A total of 1,068 contigs (0.72% of the WA contigs with BLASTx hits) had BLASTx hits to *Buchnera* sequences. An additional 1,058 contigs (1.78% of the contigs with BLASTn hits) has BLASTn hits to *Buchnera* sequences. Only 91 (20 from BLASTx and 71 from BLASTn) contigs from the gut transciptome were derived from *Buchnera* sequences and most of these were molecular chaperone sequences (e.g. GroEL), or rRNA genes. Analysis of the BLAST and annotation data for the 1,068 contigs identified by BLASTx resulted in identification of 602 non-redundant hits out of the 1,068 top hits obtained from BLASTx (Table 6, Table S2). A total of 334 distinct protein types were found from the non-redundant hits (Table S3), indicating that transcripts for more than half of the *Buchnera* genes were present in the WA aphid transriptome. Among the non-redundant top BLASTx hits, 41.2% showed homology to sequences of *Buchnera* associated with the spring grain aphid (also known as the greenbug), *Schizaphis graminum* (*Buchnera aphidicola* str. Sg), 22.2% to sequences of *Buchnera* associated with the pea aphid (*Buchnera aphidicola* str. 5A, str. Tuc7, str. LSR1 APS, JF98, and JF99), and the rest to *Buchnera* sequences from other aphid species. This result indicates that the *Buchnera* strain in the soybean aphid has diverged and is more closely related to that in the spring grain aphid, consistent with the phylogenetic relatedness of the host species: *Aphis glycines* and *Schizaphis graminum* belong to the tribe Aphidini while the pea aphid belongs to the tribe Macrosiphini.

**Table 6:** Summary of annotation of *Buchnera* sequences from whole soybean aphid transcriptome.

Number of non-redundant *Buchnera* hits with BLASTx	602
*Buchnera* of pea aphid, *Acyrthosiphon pisum*	134 (22.2%)
*Buchnera* of *Schizaphis graminum*	248 (41.2%)
*Buchnera* of *Acyrthosiphon kondoi*	107 (17.8%)
* Buchnera* of other aphids	113 (18.8%)
Distinct protein hits	329
Number of contigs that hit *Buchnera* with BLASTn	1,058
Distinct Inter Pro (Protein signatures)	39
EC (enzymes)	121
GO-terms	35

**Figure 2:**
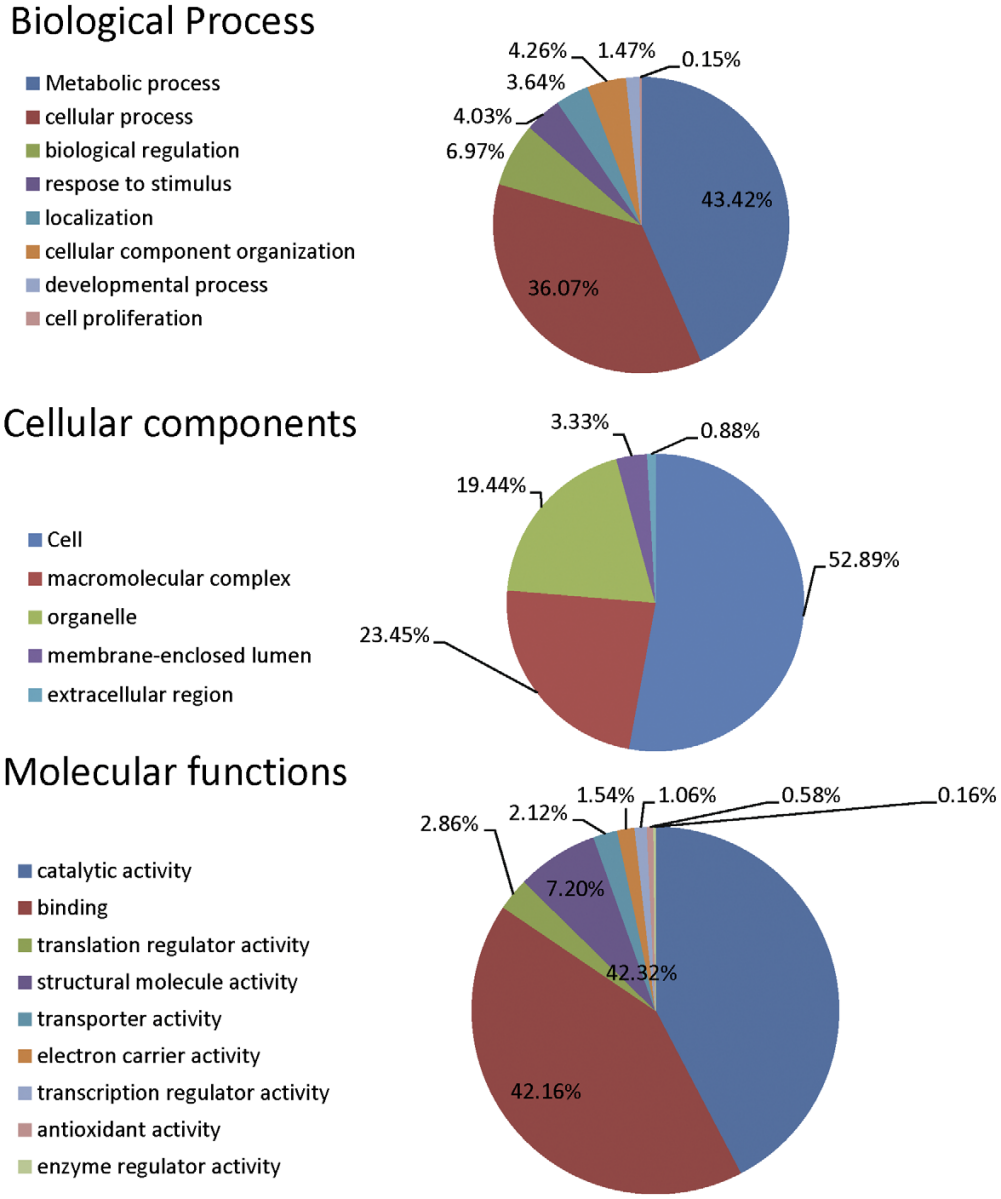
Distribution of *Buchnera* sequences by gene ontology. (GO: level 2; filtered by sequence number cutoff  = 5) for biological processes, cellular components, and molecular functions.

Gene annotation revealed that 43.4% of the *Buchnera* genes identified contain motifs that function in metabolic processes and 36% have a role in cellular processes (Figure 2). In molecular functions, 42% have catalytic activity and 42% are predicted to function in binding (Figure 2). The most highly expressed bacterial genes are the essential genes encoding ribosomal, cell division and chaperone/protease proteins [39], many of which were identified in the soybean aphid *Buchnera* transcripts. For instance, we identified the transcripts of 27 50S ribosomal protein L and 20 30S ribosomal protein S (Table S2), which were 69.2% of the annotated 50S ribosomal protein L and 74.1% of annotated 30 S ribosomal protein S from the *Buchnera* associated with the pea aphid (str. 5A and APS). We also identified eight transcripts related to cell division functions (*MInC*, Dand *E*, *FtsA, H, J W* and *Z*) and chaperone/heat shock proteins (e.g. *dnaJ, dnak, groEL, groES, HtpX, htpG, hscA, hslU*).

### *Wolbachia* is a potential secondary endosymbiont in the soybean aphid

In addition to the primary endosymbiont *Buchnera*, aphids often harbor facultative or secondary endosymbionts in their hemolymph, bacteriocytes and/or reproductive tissues [40]. Several different secondary symbionts have been identified in aphids [41], [42], with the most common species being *Serratia symbiotica*, *Hamiltonella defense*, and *Regiella insecticola* [43]. A recent study on the symbiotic bacteria of soybean aphids isolated from Illinois, USA, failed to find the secondary endosymbionts that are commonly found in aphids: PCR evidence was presented for the presence of *Arsenophonus*, a symbiont of whiteflies (Hemiptera: *Aleyrodidae*) [20]. Transcript sequence for soybean aphids isolated from Ohio, USA provided evidence for the presence of *H. defense*, which is closely related to *Arsenophonus* [27]. In searching for the secondary symbionts of soybean aphids isolated in Iowa, no significant hits were obtained by BLASTx or BLASTn to *Serratia, Hamiltonella*, or *Regiella*. However, contigs of *Arenophonus* 16S RNA were identified. PCR detection by using secondary symbiont universal 16–23S primers [44] confirmed the presence of *Arenophonus* in our soybean aphid colony (data not shown). In addition, we identified two contigs with BLASTx and 65 contigs with BLASTn, ranging from 100–771 nt in size, with similarity to *Wolbachia* sequences. *Wolbachia* is an obligatory intracellular α-proteobacterium detected in parasitic nematodes (filarial worms), mites and many insects including aphids [45]. *Wolbachia* sequences have been detected in multiple aphid species including *Toxoptera citricida*, *Aphis cracivora, Cinara cedri* and *Sitobion miscanthi* [21], [22], [41], [46], [47]. Table 7 lists the 15 contigs with the highest similarity to *Wolbachia* sequences. The corresponding contig sequences (WS1–WS15) are listed in Sequence data S1. WS1 and WS2 identified by BLASTx have homology to WwAna1270 and Scaffold protein (NifU) of *Wolbachia*, respectively. Most of the contigs identified by BLASTn are similar to either 16S or 23S ribosomal RNA with high levels of similarity (92–100%). In total, 1,070 nt of the 16S rRNA (71% of the 1,505 nt 16S rRNA of the *Wolbachia* wRi strain) and 1,686 nt of the 23S rRNA (76% of the 2,746 nt 23S of the *Wolbachia* wRi strain) were assembled into the contigs. Interestingly and consistent with previous reports [21], the 16S rRNA- like sequences of the soybean aphid-derived contigs appear to be quite diverse: The top hits of the 16S rRNA contigs were from various *Wolbachia* strains, including strains detected in filarial nematodes (*Brugia sp*.and *Dirofilaria immitis*), a mite (*Bryoba*), the Asian citrus psyllid, *Diaphorina citri* and the aphid *Cinara cedri*. 16S rDNA is commonly used for identification and classification of *Wolbachia* strains [41], [47]. The diversity of the *Wolbachia* 16S rRNA in the soybean aphid transcriptome may reflect co-infection of the soybean aphid with multiple *Wolbachia* strains, as observed in *Drosophila* [48] and the wheat aphid, *Sitobion miscanthi* [47].

**Table 7:** Wolbachia sequences identified in the soybean aphid transcriptome.

Contigs	Seq_length	Seq_description	Hit_ACC	E-value	Alig_length	Positive	Identity (%)
WS1	771	protein *WwAna1270*, *Wolbachia* endosymbiont of *Drosophila melanogaster*	ZP_00373202	5.00E-17	42 (AA)	39 (AA)	93
WS2	154	Scaffold protein, *NifU* domain protein, *Wolbachia* sp. wRi	ZP_00373458 YP_001976001	3.00E-25	51 (AA)	49 (AA)	96
WS3	225	strain trs of *Brugia* complete genome	AE017321	1.31E-86	226	209	92
WS4	129	*Dirofilaria immitis* strain dax 16s rRNA partial seq	AF487892	1.12E-58	129	129	100
WS5	178	*Wolbachia* endosymbiont of *Diaphorina citri* isolate sz 16s rRNA partial seq	GU565892	2.18E-82	178	176	98
WS6	343	*Culex quinquefasciatus* pel strain *W*pip complete genome	AM999887	1.42E-164	343	336	97
WS7	270	*Cinara cedri* 16srRNA partial seq	AY62043	3.33E-133	270	269	99
WS8	193	*Bryobia* v vidr-2008 strain ita11 16s rRNA partial seq	EU499316	5.31E-78	193	182	94
WS9	139	*Wolbachia* (from New *Caledonia*) 23s rDNA	X65683	3.54E-59	138	135	97
WS10	152	*Pentastiridius leporinus* partial 16s rRNA gene	FN428797	1.93E-69	152	151	99
WS11	297	*Wolbachia* sp. wRi, 23S rRNA	CP001391	2.33E-156	297	295	99
WS12	271	*Wolbachia* sp. wRi, 23S rRNA	CP001391	4.00E-99	224	216	96
WS13	673	*Wolbachia* sp. wRi, 23S rRNA	CP001391	0.00E+00	673	656	97
WS14	771	*Wolbachia* sp. wRi, 23S rRNA	CP001391	0.00E+00	771	748	97
WS15	107	*Onchocerca Wolbachia* seq fragment ow3	CU062463	7.56E-47	107	107	100

Sequences are provided in Supporting Information, Sequence Data S1

**Figure 3:**
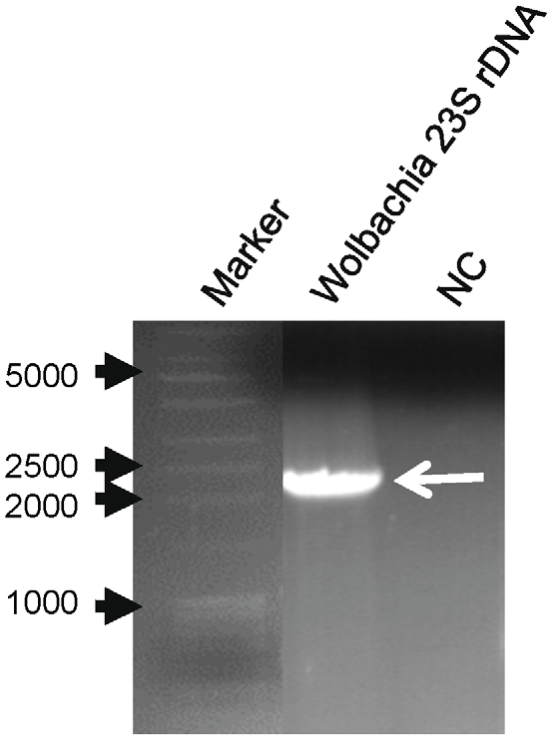
PCR detection of *Wolbachia* 23S rDNA from the soybean aphid. Markers, 1 kb DNA ladder (Fisher). NC, negative control (no template). Arrow indicates PCR product of the expected size (2.1 kbp).

In contrast to the diversity of 16S rRNA sequences, the top hit for the *Wolbachia* 23S rRNA was from *Wolbachia* sp. wRi, an endosymbiont of *Drosophila simulans*. The second hit of 23S rRNA was from strain *W*mel isolated from D. *melanogaster*. The sequences of the soybean aphid *Wolbachia* 23S rRNA contigs and the 23S rRNA of *W*mel differed only slightly from those of strain wRi, indicating that the strain of *Wolbachia* in soybean aphid may belong to *Wolbachia* group A [47]. To verify the presence of *Wolbachia* in the Iowa isolate of the soybean aphid, primers were designed based on the contig sequences to amplify 23S rDNA (Table S4). A single DNA band of the expected size (2,102 bp) was observed (Figure 3). The PCR fragment was isolated from the gel and sequenced. The sequences (two non-overlapping sequences of 915 and 1,093 bp) were subjected to BLASTx analysis with the NCBI nr database. The top five hits were all *Wolbachia* 23S rDNA sequences with the top hit being to the wRi strain, with 96% and 97% identity to the 915 and 1,093 bp fragments respectively (Sequence data S2). We also designed primers to amplify *Wolbachia Fts*, *Wsp* (two different reverse primers; Table S4) and 16S rDNA genes. Similar to previous efforts to amplify *Wolbachia* sequences from aphids [21], no product was generated by PCR using *Fts* and *Wsp* primers. Primers that were designed for amplification of 16S rDNA based on the contigs that hit the 16S rDNA of *Wolbachia*, resulted in amplification of *Buchnera* 16S rDNA.

It is important to note that there is a precedent for lateral transfer of *Wolbachia* sequences into host genomes, with *Wolbachia* genome fragments encoding multiple genes present in a host beetle [49], transfer of genome segments into the nematode *Onchocerca* [50], [51], transfers into the genomes of four insect and four nematode species, including one case of transfer of almost the entire *Wolbachia* genome [52], and transfer of *Wolbachia* genes into the genome of the tse-tse fly [53]. Hence, confirmation of the presence of *Wolbachia* in the soybean aphid and in other aphid species by using techniques other than transcript and PCR-based methods is required.

Based on the secondary endosymbionts described for soybean aphids isolated from Illinois (*Arsenophonus*) [20], Ohio (*H. defensa*) [27] and Iowa (*Arsenophonus*, *Wolbachia*), the secondary endosymbionts of the soybean aphid vary with geographical location.

### Full-length soybean aphid gene sequences

To investigate the feasibility of using RNA-seq for discovery of full-length genes, we looked for the transcript sequences for homologs of three types of genes that are relevant to potential novel soybean aphid management strategies: *C002*, a salivary gland (SG) gene which is essential for aphid feeding on the host plant [24], and two proteins that are putative secondary receptors for *Bacillus thuringiensis* Cry toxins: membrane alanyl aminopeptidase N (APN) and alkaline phosphatase (ALP)[54]. *apn* transcripts were abundant in the soybean aphid transcriptome, while the putative *C002* and *alp* transcripts were moderately expressed.

C002 is a 219 amino acid (aa) peptide, which was originally discovered from the pea aphid SG EST library. *C002* was primarily expressed in the SG of the pea aphid, but transcripts of *C002* were also detected in the gut at a level of 1% that in the SG [24]. This protein was predicted from the pea aphid genome as a hypothetical protein (XP_001948358.2, LOC100167863). By conducting local BLAST analysis with the *C002* sequence, we identified a full-length copy of the putative *C002* homolog (see Figure S1; [GenBank: JN135246]) from a single contig assembled from the WA reads with about 43-fold coverage, and a partial *C002* sequence was assembled from the gut Illumina reads, reflecting the lower expression of *C002* in the gut. The putative soybean aphid C002 is 214 aa, 5 aa shorter than that of the pea aphid C002. Alignment of the soybean C002 homolog with the pea aphid C002 showed less than 50% sequence identity at the protein level (Figure S1b). C002 is secreted into the host plant and plays an important role in feeding, and hence may be involved in host plant selection [24]. The lower identity between the soybean aphid and pea aphid C002 may reflect the differences in the host plant preferences of the two species and selection for divergent protein sequences to deal with some aspect of survival on the host plant. Functional analysis is required to confirm that silencing of this gene in the soybean aphid has similar effects to those reported for the pea aphid [25].

More than 10 APN- and six full-length ALP-like genes, including isoforms and transcript variants, were predicted and annotated from the pea aphid genome. The sizes of the APN and ALP of the pea aphid were between 524–1039 aa and 513–565 aa, respectively. To identify APN-like and ALP-like genes from the soybean aphid, we analyzed BLASTx data and identified > 600 hits with contigs from the gut and 247 hits with contigs from WA against pea aphid APN genes. However, only 71 of the contigs were >1000 nt with the longest contig being >3200 nt. From these contigs, we found only two with the predicted full-length APN sequences. For ALP genes, 200 soybean aphid contigs were similar to the ALP genes of the pea aphid. The longest ALP contig was 1,830 nt, and two putative full-length ALP genes were identified. Notably, none of these predicted full-length genes were assembled by using the same *k* and *C* combinations. On further analysis of the contigs, one additional APN and one additional ALP full-length genes were identified by aligning the contigs and re-assembling the overlapping fragments. In addition, fragments of APN and ALP genes were also identified. To verify the presence of the full-length genes in the soybean aphid, RT-PCR was carried out to amplify the potential full-length APN transcripts (see Table S4 for primer sequences). cDNA was generated with polyT oligo, and primers specific to the four APN genes were used for PCR. cDNA of the four APNs of the correct sizes were successfully detected. Sequencing of the PCR-amplified APN4 cDNA showed that only 10 nucleotides (0.03%) differed from the APN4 sequences generated by the Illumina reads. The sequences for soybean aphid APN and ALP were submitted to GenBank [GenBank: ALP1 JN135238; ALP2 JN135239; ALP3 JN135240; ALP4 JN135241 (partial sequence); APN1 JN135242; APN2 JN135243 (partial sequence); APN3 JN135244; APN4 JN135245].

**Figure 4:**
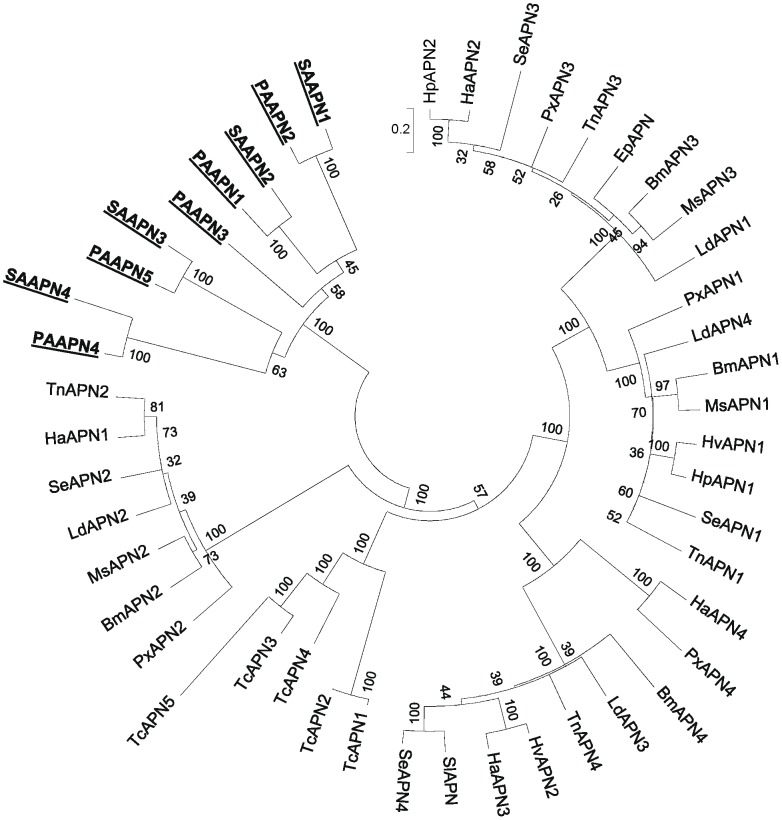
Phylogenetic relatedness of soybean aphid aminopeptidese N (APN) derived from the gut transciptome with lepidopteran APN. The phylogenetic tree drawn to scale was generated by using the maximum-likelihood method using MEGA 5.0 with a bootstrap value of 500. Soybean aphid (SA), and pea aphid (PA) sequences are boxed. GenBank accession numbers: *Bombyx mori*: BmAPN1, AAC33301, BmAPN2, BAA32140, BmAPN3, AAL83943, BmAPN4, BAA33715; *Epiphyas postvittana*, EpAPN, AAF99701; *Helicoverpa armigera*, HaAPN1, AAW72993, HaAPN2, AAN04900, HaAPN3, AAM44056, HaAPN4, AAK85539; *Helicoverpa punctigera*: HpAPN1, AAF37558, HpAPN2, AAF37560; *Heliothis virescens*: HvAPN1, AAF08254, HvAPN2, AAK58066; *Lymantria dispar*: LdAPN1, AAD31183, LdAPN2, AAD31184, LdAPN3, AAL26894; LdAPN4, AAL26895; *Plutella xylostella*: PxAPN1, AAB70755, PxAPN2, CAA66467, PxAPN3, AAF01259, PxAPN4, CAA10950; *Manduca sexta*: MsAPN1, CAA61452, MsAPN2, CAA66466, MsAPN3, AAM13691, MsAPN4, AAM18718; *Spodoptera exigua*: SeAPN1, AAP44964, SeAPN2, AAP44965, SeAPN3, AAP44966, SeAPN4, AAP44967; *Spodoptera litura*: SlAPN, AAK69605; *Trichoplusia ni*, TnAPN1, AAX39863, TnAPN2, AAX39864, TnAPN3, AAX39865, TnAPN4, AAX39866; *Tribolium castaneum*: TcAPN1, EEZ99298; TcAPN2, XP_001812439; TcAPN3, XP_972987; TcAPN4, XP_972951; TcAPN5, XP_973022; the pea aphid, *A. pisum*: PAAPN1, NP_001119606, PAAPN2, XP_001946370, PAAPN3, XP_001946754, PAAPN4, XP_001948442 PAAPN5, XP_001948350, SAAPN1 JN135242; SAAPN2, JN135243; SAAPN3, JN135244, SAAPN4, JN135245.

**Figure 5:**
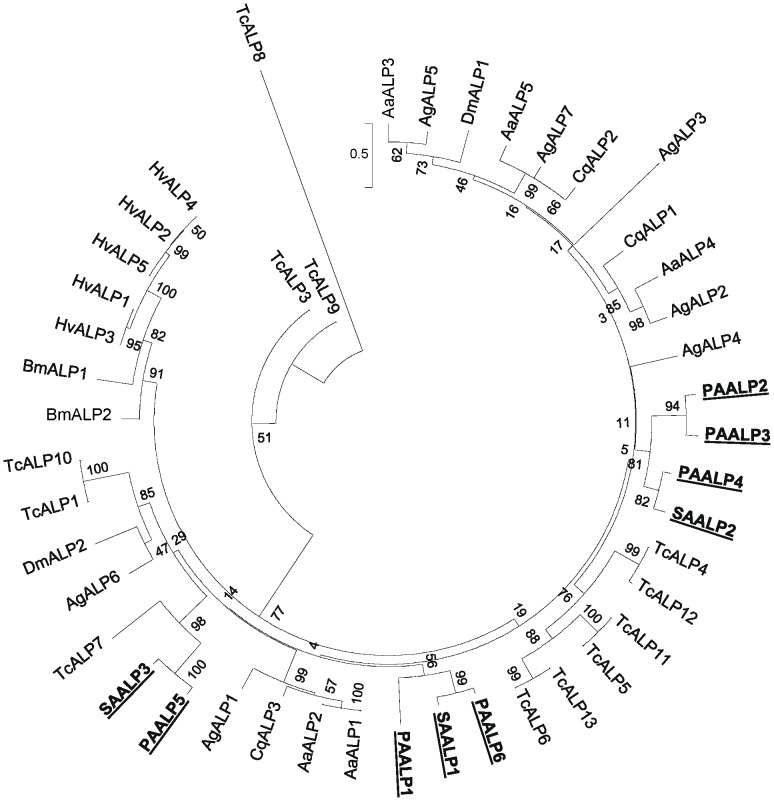
Phylogenetic analysis of insect alkaline phosphatases (ALP). Phylogenetic tree drawn to scale for the soybean aphid (SA), pea aphid (PA) ALP, and mosquito (*Aedes aegypti*, Aa; *Anopheles gambiae*, Ag; *Culex quinquefasciatus*, Cq) lepidopteran (*Bombyx mori*, Bm; *Heliothis virescens*, Hv), *Drosophila melanogaster* (Dm) and *Tribolium castaneum* (Tc) ALPs. Soybean aphid (SA), and pea aphid (PA) sequences are boxed. GenBank accession numbers: AaALP1, XP_001663478, AaALP2, XP_001649092,AaALP3 XP_001648006, AaALP4, XP_001663538, AaALPXP_001663535; AgALP1, XP_313890, AgALP2, XP_001688180,AgALP3, XP_316433, AgALP4, XP_308522 AgALP5, XP_321411, AgALP6,XP_314561, AgALP7, XP_309345BmALP1, NP 001037536, BmALP2, NP_001036856; CqALP1, XP_001842934, CqALP2, XP_001842932; DmALP1, NP_001034040, DmALP12, NP_524601; HvALP1, ACP39712, HvALP2, ACP39713, HvALP3, ACP39714, HvALP4, ACP39715, HvALP5, ABR88230; TcALP1, XP_975050, TcALP2, XP_973094, TcALP3, EFA08950, TcALP4, EFA08951, TcALP5, EFA08952, TcALP6, XP_968925, TcALP7, EEZ99048, TcALP8, EEZ99048, TcALP9, EEZ99049, TcALP10, EFA01926, TcALP11, XP_971418, TcALP12, XP_971358; TcALP13, XP_971482; The pea aphid, *A. pisum*: PAALP1, XP_001944129, PAALP2, XP_001943536, PAALP3, XP_001943259, PAALP4, XP_001943482, PAALP5, XP_001943355 PAALP6, XP_001943535; SAALP1 JN135238; SAALP2, JN135239; SAALP3, JN135240.

APN and ALP are important receptors for Cry toxins derived from the bacterium *Bacillus thuringiensis* (Bt) [54]. As Cry toxins are not particularly effective against aphids [26], we sought to address whether divergence of the putative receptor proteins could contribute to the low toxicity. Phylogenetic analysis of APN sequences between aphids (the soybean aphid and the pea aphid) and lepidopteran species [55] showed that aphid APNs are distinct from other classes of insect APN and form their own clade (Figure 4). The aphid ALPs were compared with those derived from mosquito, lepidopteran species, *Drosophila* and *Tribolium castaneum*. The ALPs of aphids divide into three groups (Fig 5). Divergence of the putative Bt receptor proteins in aphids may contribute to the relatively low toxicity of Bt-derived toxins against aphids [26]

## Conclusions

In this study, we analyzed ∼400,000 contigs generated by *de novo* assembly from RNA-seq reads of the soybean aphid gut and WA transcriptomes. The use of multiple sets of contigs with varying k and C parameters, and BLAST analysis significantly increased the number of transcripts identified, and the acquisition of full length gene sequences. This can be explained by the fact that contigs with fewer reads in the data set contain valuable transcript information that would otherwise be excluded when a higher coverage cutoff threshold is used.

Annotation of the contigs by BLAST allowed for identification of almost half of the pea aphid gene homologs from the soybean aphid transcriptome, and more than 50% of the *Buchnera* transcripts. This approach also allowed for identification of full-length aphid genes and the discovery of a potential new secondary endosymbiont, *Wolbachia* from the soybean aphid. Our results significantly increase the genomic resources available for the soybean aphid, and demonstrate use of the Multiple-*k*/Multiple-*C* methodology on a short read sequence data set for enhanced data mining. These results highlight the potential of RNA-seq for genomics and functional genomics studies on organisms for which genomic sequence data are not available, and extend the potential utility to endosymbiont transcriptomes. This work will provide the foundation for future analyses of soybean aphid biotype formation, the role of facultative endosymbionts in aphid adaptation, and for development of novel technologies for soybean aphid management.

## Materials and Methods

### Insect rearing

A colony of soybean aphids, *Aphis glycines* Matsumura, was established from aphids collected in soybean fields in Iowa. The colony was maintained on soybean *Glycine max* (Variety 92M91, Pioneer Hi-Bred International, Inc. Johnston, IA) at 24±1°C with a 12 h light/12 h dark cycle and only produced viviparous parthenogenetic females

### RNA isolation and transcriptome sequencing

Three RNA samples were prepared, one from aphid guts, and two from whole aphids. For isolation of RNA from soybean aphid guts, the entire digestive tract was removed under a dissection microscope (Nikon SMZ 1500) from fourth and fifth instar nymphs, with approximately one-tenth of the sample derived from adults. Approximately 2,000 guts were pooled and stored in TRIzol reagent (Invitrogen). RNA was isolated and purified according to the TRIzol protocol. Total RNA was isolated from whole aphids (WA) (300 mg, all instars, winged and wingless nymphs, and adults).

Two steps of poly-A RNA purification were conducted for two samples (WA and gut) using oligo (dT) magnetic beads and further processed according to Illumina protocols. For the second WA sample, a single polyA purification step was carried out, resulting in increased representation of *Buchnera* sequences within the transcriptome. RNA integrity was confirmed using a 2100 Bioanalyzer (Agilent Technologies). The purified RNA was used to prepare samples for sequencing by using the Illumina truSeq RNA sample preparation kit. Sequencing on an Illumina GAII sequencing platform (Illumina Corporation) at the Iowa State University DNA Facility resulted inapproximately 8 million single-end reads for each lane, mostly 75 nt in length for each sample. In total, approximately 24 million reads were obtained. Adapter sequences and low quality sequences were removed prior to further analysis.

### Bioinformatics

Aphid transcriptome sequences were mapped to the draft 207 genome (Acyr_1.0) of the pea aphid, *A. pisum* (http://www.aphidbase.com/aphidbase) [56] using the Eland (Illumina Inc.) and MAQ programs (http://maq.sourceforge.net/) with a maximum of 2 mismatches for Eland and 3 mismatches for MAQ. The Illumina reads were assembled using the Velvet assembler (1.0)[28], run on an Apple Mac Pro computer with 8-core Two 2.93GHz Quad-Core Intel Xeon/16GB RAM. Assembly was performed by using various combinations of *k* and *C* parameters and according to the program manual. Use of the multiple-*k* method significantly improves assembly efficiency [29]. By combining the multiple-*k* and multiple-*C* methods for assembly, followed by depleting redundant contigs, the numbers of assembled contigs was greatly increased. The selected contigs with a length cutoff of 100 nt were used for annotation by searching against the GenBank non-redundant database (including the *A. pisum* genome Acyr_2.0) using BLASTx algorithms (Number of BLAST hits  = 1 (return only top hit); minimum e-value  = 1.0-E3, BLAST model: QBLAST-NCBI; HSP length cut off  = 33; lower capacity filer  =  yes). Contigs without BLASTx hits were then annotated by using BLASTn algorithms using similar parameter settings to those used for BLASTx analyses. For optimal assignment of annotation quality and BLAST result analysis, only the top hits from BLAST were used for further data analyses. Gene Ontology (GO) annotation was conducted by using the Swiss-Prot database (http://www.uniprot.org/) and the protein signatures were annotated by using InterProScan [57]. All annotation programs were performed using the BLAST2GO platform [58]; http://www.blast2go.org/start_blast2go For annotation of combined contig sets, the contigs were purged for removal of redundant sequences using CD-HIT [30]; http://weizhong-lab.ucsd.edu/cd-hit/

The data sets are available at the NCBI Short Read Archive (SRA) with accession number: SRA038331.

### Full length gene assembly and data analysis

For assembly of putative full length soybean aphid genes, contigs (> = 300 nt) were aligned using BioEdit 7.0.9: http://www.mbio.ncsu.edu/BioEdit/bioedit.html The assembled cDNA fragments were translated and aligned to the genes of the pea aphid. The putative full length genes were then used for phylogenetic analysis. The multiple sequence alignments and phylogenetic trees (maximum-likelihood trees) were generated using MEGA 5.0 with a bootstrap value of 500 [59].

### Assessment of relative transcript abundance

The depth of reads assembled into a contig was used to assign relative transcription levels within the transcriptome. Reads were mapped to the reference contigs using MAQ. The depth of mapping was recorded and the 50 contigs with the highest number of reads were analyzed.

qRT-PCR was used to validate the relative expression levels as determined by RNASeq, of APN3, APN4, and two contigs with low transcript abundance (Few Transcripts, FT1, FT2). Total RNA from soybean aphid guts (0.5 mg) was isolated by using Trizol reagent (Invitrogen) according to the manufacturer's directions. Precipitated RNA was resuspended in DEPC-treated, autoclaved water and stored at −80°C until further use. qRT-PCR was performed in two steps: In the first step, a 20 µl RT reaction was set up using 5 µg of soybean aphid gut total RNA, oligo dT12-18 primers and Superscript reverse transcriptase to synthesize the first strand cDNA according to the recommended protocol (Invitrogen). qRT-PCR primers for all four genes (*apn3*, *apn4*, *FT1*, and *FT2*: See Table S4 for primer sequences) were tested by PCR to confirm amplification of a single product of the correct size (200 bp). Twenty µl qRT-PCR reactions to amplify all four genes and GAPDH (internal control [60]) were set up in a 96 well plate using IQ Syber Green supermix (Bio-Rad). Two sets of negative controls, the no template control and the total RNA template (to control for contamination with genomic DNA) were set up for each primer pair. For amplification of sequences from all five genes, PCR reactions were performed using the following thermal cycle conditions: 95°C for 3 min, followed by 95°C for 15 s, 52°C for 30 s, and 72°C for 30 s for 40 cycles. PCR reactions were performed with two biological and three technical replicates, and analyzed on a Bio-Rad iCycler™ iQ Optical system using Software Version 3.0a. Values for relative transcript abundance for each of the four genes were calculated and normalized with reference to transcript abundance for the internal control. The relative expression levels of the four genes were compared by one-way ANOVA.

### Confirmation of *Wolbachia* 23S rDNA sequence

Total DNA was extracted from 50 soybean aphids using DNAzol ® (Invitrogen) according to the manufacturer's protocol, and dissolved in nuclease free water. The primers (Table S4) were designed based on the assembled contig from the soybean aphid transcriptome that had homology to the nearest 5′ and 3′ ends of the *Wolbachia* 23S rDNA. PCR was performed using Choice Taq ™ DNA Polymerase and with 1 cycle of 94°C for 2 min, 35 cycles of 94°C for 30sec, 53 or 55°C (see Table S4) for 30sec, 72°C for 3 min and 1 cycle of 72°C for 5 min. The amplified PCR product (2,102 bp) was run on a 1% agarose gel. The PCR product was removed from the gel and purified using the Qiaquick gel extraction kit (Qiagen). The purified PCR product was eluted in nuclease free water and submitted to the Iowa State University DNA Facility for sequencing using both forward and reverse primers.

## Supporting Information

Table S1 **BLASTn hits for contigs identified by BLASTx to have similarity to barley sequences.** The top two BLASTn hits are indicated.
(XLSX)

Table S2 **Non-redundant hits of *Buchnera* genes by BLASTx.** The list contains the contig ID, length, and BLAST hit descriptions for 602 sequences.
(XLSX)

Table S3 ***Buchnera*** **proteins identified from the soybean aphid transcriptome.** The list contains the contig ID, contig length, and 334 corresponding protein and genes names, and descriptions.
(XLSX)

Table S4 **Primer sequences.** Sequences are provided for primers used for PCR amplification of *apn-3, apn-4, FT1, FT2* and *Wolbachia* (*ftz, wsp, 16S, 23S*) gene fragments.
(XLSX)

Figure S1 **Soybean aphid putative homolog of salivary protein C002.** A. Sequence of the putative pea aphid C002 homolog from the soybean aphid; B. Clustal W alignment of the C002 amino acid sequences from the pea aphid and the soybean aphid.
(PDF)

Sequence data S1 **Sequences from soybean aphid transcriptome contigs derived from the secondary endosymbiont *Wolbachia***. Sequences were derived from the whole aphid transcriptome (WA). Fifteen sequences are provided (WS1-WS15).
(PDF)

Sequence data S2 **Additional evidence for the presence of *Wolbachia* in the soybean aphid.** The Wolbachia 23S rDNA sequences derived from the soybean aphid were PCR-amplified and sequenced. The alignment of the soybean aphid (SA) PCR-amplified sequence with the sequence of *Wolbachia* sp. wRi (wRi) is provided.
(PDF)
