# Supplementary material for: Deep Sequencing of the Transcriptomes of Soybean Aphid and Associated Endosymbionts
Source: PLoS One. 2012 Sep 12;7(9):e45161. doi: 10.1371/journal.pone.0045161 (PMC3440339; doi:10.1371/journal.pone.0045161)
Supplement: Figure S1 — Soybean aphid putative homolog of salivary protein C002. A. Sequence of the putative pea aphid C002 homolog from the soybean aphid; B. Clustal W alignment of the C002 amino acid sequences from the pea aphid and the soybean aphid. (PDF) [file pone.0045161.s005.pdf]

A

```
>Soybean aphid c002 homolog
3      CCG CTC TTC CGA TCT ATC GCC GTG TAG CAC AGG ATA GCG ATT ATA   47
48     AAC AGT ATG GGA CGT TAC CAA TTA TAT GTG GCC GTC ATG GCA ATA   92
                M   G   R   Y   Q   L   Y   V   A   V   M   A   I    13
93     TCT TTT AGC TTA GCC GTA ATA CAG AAA GCT AGT TGT GCT GGT GAG   137
14      S   F   S   L   A   V   I   Q   K   A   S   C   A   G   E    28
138    TCT GAC GCC AAC CCC ACT ACC GAA CAA TAC ATT GAG TCA AAA GAC   182
29      S   D   A   N   P   T   T   E   Q   Y   I   E   S   K   D    43
183    GAA TTA GAA ATG GAG CAT CAT CAG TGT GAT GAA TAC AAA TCG AAA   227
44      E   L   E   M   E   H   H   Q   C   D   E   Y   K   S   K    58
228    ATC TGG AAT AAG GCA TTT AGC AAC CCA GCG GCT ATG CAA CTG ATG   272
59      I   W   N   K   A   F   S   N   P   A   A   M   Q   L   M    73
273    GAC GTA GTG CTT GAA ACA GCT AAG GAA TTG GGA ACC AAT GAC GTG   317
74      D   V   V   L   E   T   A   K   E   L   G   T   N   D   V    88
318    TGC TCA GAC ACG ATT CGG GTC TTG TCT AAC TTC ATC GAT GTG ATG   362
89      C   S   D   T   I   R   V   L   S   N   F   I   D   V   M    103
363    GTC ACC AAT CAG AAC TCC CAC TAC TCG GTG GGA ATG TTG GTA AAG   407
104     V   T   N   Q   N   S   H   Y   S   V   G   M   L   V   K    118
408    ATG CTG GCA TTC ATT GCG AGA GAA GCG GAT ACG ACG TCG GAC AAA   452
119     M   L   A   F   I   A   R   E   A   D   T   T   S   D   K    133
453    TTC AGA GAT ACA AAG AAG GTG TTC GAT CGC ATC GTA AAA AAT GCT   497
134     F   R   D   T   K   K   V   F   D   R   I   V   K   N   A    148
498    GAT ATC CGT GAC TAT ATC AGA AAC ACG GTC TCC CAG GTT GTC GAC   542
149     D   I   R   D   Y   I   R   N   T   V   S   Q   V   V   D    163
543    TTG CTC AAG TTA CCC GTG ATG AGA AAT CGA TTA GCC AGA GTG TTT   587
164     L   L   K   L   P   V   M   R   N   R   L   A   R   V   F    178
588    AAA GCC TTT GAG AGT TTG TAT AAT CCA TCC AAA AAA CCA GCA AAT   632
179     K   A   F   E   S   L   Y   N   P   S   K   K   P   A   N    193
633    GAG CAA GCA GAG GAT TAT GGG ACT AAC CAA CAC TCC TTC CAA AAT   677
194     E   Q   A   E   D   Y   G   T   N   Q   H   S   F   Q   N    208
678    AGC TAT GGG TAC CAT GAA TAA GGT TGA AAA TAT GTT TCC AAA TTT   722

209     S   Y   G   Y   H   E   *                                    214

723    CTA ATA AAC ACG                                               734
```

B

```
CLUSTAL W (1.81) multiple sequence alignment


c002-pea_aphid          MGSYKLYVAVMAIAIAVVQEVRCDWS----AAEPYDEQEEASVELPMEHRQCDEYKSKIW
c002-soybean_aphid      MGRYQLYVAVMAISFSLAVIQKASCAGESDANPTTEQYIESKDELEMEHHQCDEYKSKIW
                        ** *:********::::.   :.. :    *  . ::  *:. ** ***:**********

c002-pea_aphid          DKAFSNQEAMQLMELTFNTGKELGSHEVCSDTTRAIFNFVDVMATNQNAHYSLGMMNKML
c002-soybean_aphid      NKAFSNPAAMQLMDVVLETAKELGTNDVCSDTIRVLSNFIDVMVTNQNSHYSVGMLVKML
                        :*****  *****::.::*.****:::***** *.: **:***.****:***:**: ***

c002-pea_aphid          AFIIREVDTTSNKFKETKEVFERIAKTPEIRDYIKHTTARTVDLLKEPVIRGRLFKVVKA
c002-soybean_aphid      AFIAREADTTSDKFRDTKKVFDRIVKNADIRDYIRNTVSQVVDLLKLPVMRNRLARVFKA
                        *** **.****:**::**:**:**.*..:*****::*.::.***** **:*.** :*.**

c002-pea_aphid          FEGLIKPSENEELVKQRLKRITNAPAKMAMGAINKFGSFLRRF
c002-soybean_aphid      FESLYNPSKKPANEQAEDYGTNQHSFQNSYGYHE---------
                        **.* :**::    : .    .: . : : *  :
```

Figure S1
