# Supplementary material for: Deep Sequencing of the Transcriptomes of Soybean Aphid and Associated Endosymbionts
Source: PLoS One. 2012 Sep 12;7(9):e45161. doi: 10.1371/journal.pone.0045161 (PMC3440339; doi:10.1371/journal.pone.0045161)
Supplement: Sequence data S1 — Sequences from soybean aphid transcriptome contigs derived from the secondary endosymbiont Wolbachia. Sequences were derived from the whole aphid transcriptome (WA). Fifteen sequences are provided (WS1-WS15). (PDF) [file pone.0045161.s006.pdf]

Sequence Data S1

Soybean aphid contig sequences that hit Wolbachia genes

>WS1|hypothetical protein WwAna1270 >ref|ZP_00373202.1| hypothetical protein WwAna1270 [Wolbachia endosymbiont of Drosophila anaanssae]
CGCTACTCCCTATTTTGTGCCACCTGCCAATAGTTGCCTAAAAGCAGGTTACCCTTCTTCCGAAG
TTACAGGTATAATTTGCCGAGTTCCTTCAACATCATTCTTTCAACACCTTAGTATACTCTACTCA
TCCACCAGTGTCGGTTTACGGTACGGCCTCATAAATATAAGTGCTATTTCCTGGAGCTTCTTTTA
AGCATAAGTCAATCCAATAAGACCTATACAAATACAAAACCCGTCACACTTAAGAGGTTTAGGAA
TATTAACCTAATTGCCATCGACTACTCCTTTACGGACTCGCCTTAGGAACCGACTAACCCTACGC
AGATTAACTTAACGTAGGAAACCTTAGATTTTTGGTGAGAGTGTTTTTCACACTCTTTTACGCTA
CTTATGTCAGCATTCTCACTTCCGATATCTCGAGTAGTCTTCTCAAACTACCTTCACAGACTTAC
GGAACGCTCCGCTACCGCGCCTATTGATCAAGATCAATAAGCACTCACATCTTCGGTATACAGCT
TTAGCCCCGGTACATTTTCAGCGCAGAAAAACTTATTTAGACAAGTCAGCTGTTACGCTTTCTTT
AAATGATGGCTGCTTCCAAGCCAACCTCCTAGCTGTAATGGTTTTTCTACTTCCTTCCCCACTTA
GCTGTAATTTTGGGACCTTAGATAGTGATCTGGGTTGTTTCCCTTTTCACCACGGACTTAGCACC
CGCAGTGTGTCTGCTGTATAATTAATTGTTGGTATTCGGAGTTTGGTTAGATTTGG

>WS2|[Wolbachia endosymbiont of Drosophila melanogaster] ref|ZP_00373458.1| FeS cluster assembly scaffold IscU [Wolbachia endosymbiont of Drosophila ananassae]
TGAACTAGAAGCAATTGCGGAACCACAACCATATGTTTTAAAACAAGCATCTTCAATGATACCTT
TCGAATTAACTTTAATTTGTAATTTCATAACATCACCACATGATGGTGCACCAACCAAACCAGTT
CCAACATGTGGATCATCCTTATCC

>WS3
ACTCGCTGACCCATTATGCAAAAGGTACGCTGTCACTCTAATATAAATTCAATACAAGAT
TGCTCTTGCAGAAAAATATAAAGCTCCAACTGTTTGTAAGCACTTGATTTCAGGATCTAT
TTCACTCCCCTCCCGGGGTTCTTTTCACCTTTCCCTCACGGTACTCGTTCACTATCGGTC
GTTAAGGAGTATTTAGGCTTGGAGGATGGTCCCCCCATATTCAAA

>WS4
TTAATTCGATGCAACGCGAAAAACCTTACCACTCCTTGACATGGAAATTATACCTATTCG
AAGGGATAGGGTCGGTTTGGCCGGATTTCACACAGGTGTTGCATGGCTGTCGTCAGCTCG
TGTTGTGAA

>WS5
AGAGATTCTGTTAGTAGTGACGAGCGAACGCGGAAAAGGCTAGTGATTTAAAAATAAGAA
TTAAAATACTCTGGAAATAGTAACCATAGAAGGTGATAGTCCTGTATAAGTAGAAAGTTT
TTAAATCCTCGAGTAGGGCGGGACACGTGAAATCCTGTTTGAATATGGGGGGACCATC

>WS6
GTTAATATTCCTAAACCTCTTAAGTGTGACGGGTTTTGTATTTGTATAGGTCTTATTGGA
TTGACTTATGCTTAAAAGAAGCTCCAGGAAATAGCACTTATATTTATGAGGCCGTACCGT
AAACCGACACTGGTGGATGAGTAGAGTATACTAAGGTGTTGAAAGAATGATGTTGAAGGA
ACTCGGCAAATTATACCTGTAACTTCGGAAGAAGGGTAACCTGCTTTTAGGCAACTATTG
GCAGGTGGCACAAAATAGGGAGTAGCGACTATTTACTAAAAACACAGGACTCTGCAAACA
CGTAAGTGGAAGTATAGGGTCTGATGCCTGCCCGGTGCTGGAA

>WS7
TAAGTCCCGCAACGAGCGCAACCCTCATCCTTAGTTACCATCAGGTTATGCTGGGGACTT

TAAGGAAACTGCCAGTGATAAACTGGAGGAAGGTGGGGATGATGTCAAGTCATCATGGCC
CTTATGGAGTGGGCTACACACGTGCTACAATGGTGATTACAATGGGCTGCAAGGTCGCAA
GGCTGAGCTAATCCTAAAAAATCATCTCAGTTCGGATTGTTCTCTGCAACTCGAGAGCAT
GAAGTTGGAATCGCTAGTAATCGTGGATCA

>WS8
GCGTAAAGAGCGCGTAGGCTGATTAGTAAGTTAAAAGTGAAATCCCAAAGCTTAACTTTG
GAATTGCTTTTAAAACTGCTAGTCTAGAGATTGAAAGAGGATAGAGGAATTCCTAGTGTA
GAGGTGAAATTCGTAAATATTAGGAGGAACACCAGTGGCGAAGGCGGTTCTCTGGGCCTT
TCCTGACGCTGAG

>WS9
CTTTCGTCTCTGCTTGGCTTGTCAGCCTTGCAGTCAGGCAAGCTTATGCCATTATACTAT
CAAGCTGATTTCCGACCAGCTCTAGCTTACCTTCGCACGCCTCCGTTACTTTTTAGGAGG
CGACCGCCCCAGTCAAACT

>WS10
TAAGTCCCGCAACGAGCGCAACCCTCATCCTTAGTTACCATCAGGTTATGCTGGGGACTT
TAAGGAAACTGCCAGTGATAAACTGGAGGAAGGTGGGGATGATGTCAAGTCATCATGGCC
CTTATGGAGTGGGCTACACACGTGCTACAATG

>WS11
TGGTGGGTAGTTTGACTGGGGCGGTCGCCTCCTAAAAAGTAACGGAGGCGTGCGAAGGTA
AGCTAGAGCTGGTCGGAAATCAGCTTGATAGTATAATGGCATAAGCTTGCCTGACTGCAA
GGCTGACAAGCCAAGCAGAGACGAAAGTCGGTCATAGTGATCCGGTGATTCTGTATGGAA
GGGTCATCGCTCAACGGATAAAAGGTACGCCGGGGATAACAGGCTGATGGTGTTCAAGCG
TTCATAGCGACGACACCGTTTGGCACCTCGATGTCGACTCATCACATCCTGGGGCTG

>WS12
TACCACCCTTCACACTTTTAATATCTAACTATGTTTCATTATCTGGAACTAGGACATTGT
ATGGTGGGTAGTTTGACTGGGGCGGTCGCCTCCTAAAAAGTAACGGAGGCGTGCGAAGGT
AAGCTAGAGCTGGTCGGAAATCAGCTTGATAGTATAATGGCATAAGCTTGCCTGACTGCA
AGGCTGACAAGCCAAGCAGAGACGAAAGTCGGTCATAGTGATCCGGTGATTCTGTATGGA
AGGGTCATCGCTCAACGGATAAAAGGTACGC

>WS13
TTAACCTTCCAGCACCGGGCAGGCGTCAGACCCTATACTTCCACTTACGTGTTTGCAGAG
TCCTGTGTTTTTAGTAAACAGTCGCTACTCCCTATTTTGTGCCACCTGCCAATAGTTGCC
TAAAAGCAGGTTACCCTTCTTCCGAAGTTACAGGTATAATTTGCCGAGTTCCTTCAACAT
CATTCTTTCAACACCTTAGTATACTCTACTCATCCACCAGTGTCGGTTTACGGTACGGCC
TCATAAATATAAGTGCTATTTCCTGGAGCTTCTTTTAAGCATAAGTCAATCCAATAAGAC
CTATACAAATACAAAACCCGTCACACTTAAGAGGTTTAGGAATATTAACCTAATTGCCAT
CGACTACTCCTTTACGGACTCGCCTTAGGAACCGACTAACCCTACGCAGATTAACTTAAC
GTAGGAAACCTTAGATTTTTGGTGAGAGTGTTTTTCACACTCTTTTACGCTACTTATGTC
AGCATTCTCACTTCCGATATCTCGAGTAGTCTTCTCAAACTACCTTCACAGACTTACGGA
ACGCTCCGCTACCGCGCCTATTGATCAAGATCAATAAGCACTCACATCTTCGGTATACAG
CTTTAGCCCCGGTACATTTTCAGCGCAGAAAAACTTATTTAGACAAGTGAGCTGTTACGC
TTTCTTTAAAGGA

>WS14
CGCTACTCCCTATTTTGTGCCACCTGCCAATAGTTGCCTAAAAGCAGGTTACCCTTCTTC
CGAAGTTACAGGTATAATTTGCCGAGTTCCTTCAACATCATTCTTTCAACACCTTAGTAT
ACTCTACTCATCCACCAGTGTCGGTTTACGGTACGGCCTCATAAATATAAGTGCTATTTC
CTGGAGCTTCTTTTAAGCATAAGTCAATCCAATAAGACCTATACAAATACAAAACCCGTC

ACACTTAAGAGGTTTAGGAATATTAACCTAATTGCCATCGACTACTCCTTTACGGACTCG
CCTTAGGAACCGACTAACCCTACGCAGATTAACTTAACGTAGGAAACCTTAGATTTTTGG
TGAGAGTGTTTTTCACACTCTTTTACGCTACTTATGTCAGCATTCTCACTTCCGATATCT
CGAGTAGTCTTCTCAAACTACCTTCACAGACTTACGGAACGCTCCGCTACCGCGCCTATT
GATCAAGATCAATAAGCACTCACATCTTCGGTATACAGCTTTAGCCCCGGTACATTTTCA
GCGCAGAAAAACTTATTTAGACAAGTCAGCTGTTACGCTTTCTTTAAATGATGGCTGCTT
CCAAGCCAACCTCCTAGCTGTAATGGTTTTTCTACTTCCTTCCCCACTTAGCTGTAATTT
TGGGACCTTAGATAGTGATCTGGGTTGTTTCCCTTTTCACCACGGACTTAGCACCCGCAG
TGTGTCTGCTGTATAATTAATTGTTGGTATTCGGAGTTTGGTTAGATTTGG

>WS15
CCCCGGTACATTTTCAGCGCAGAAAAACTTATTTAGACAAGTGAGCTGTTACGCTTTCTT
TAAAGGATGGCTGCTTCCAAGCCAACCTCCTAGCTGTAATGGTTTTT
