# Supplementary material for: Deep Sequencing of the Transcriptomes of Soybean Aphid and Associated Endosymbionts
Source: PLoS One. 2012 Sep 12;7(9):e45161. doi: 10.1371/journal.pone.0045161 (PMC3440339; doi:10.1371/journal.pone.0045161)
Supplement: Sequence data S2 — Additional evidence for the presence of Wolbachia in the soybean aphid. The Wolbachia 23S rDNA sequences derived from the soybean aphid were PCR-amplified and sequenced. The alignment of the soybean aphid (SA) PCR-amplified sequence with the sequence of Wolbachia sp. wRi (wRi) is provided. (PDF) [file pone.0045161.s007.pdf]

Sequencing Data S2

Soybean aphid Wolbachia 23S rDNA sequences

SA: wolbachia_23s_rDRNA of Soybean aphid(Forward)
WRi: Wolbachia sp. wRi, complete genome (CP001391.1, 1445873 bp)

Sequence 1 (forward sequencing of the DNA fragment) Score = 1443 bits (781), Expect = 0.0
Identities = 875/915 (96%), Gaps = 27/915 (3%) Strand=Plus/Plus

```
SA  1     GATTTAA-AATAAG-ATTAAAATACTCTGGAAATAGTAACCATAGAAGGTGATAGTCCTG  58
          ||||||| |||||| |||| ||||||||||||||||||||||||||||||||||||||||
WRi188791 GATTTAAGAATAAGAATTAGAATACTCTGGAAATAGTAACCATAGAAGGTGATAGTCCTG  188850

  59      TATAAGTAGAAAGTTTTTAAATCCTCGAGTAGGGCGGGACACGTGAAATCCTGTTTGAAT  118
          ||||||||||||||||||||||||| |||||| ||||| |||||||||||||||||||||
  188851  TATAAGTAGAAAGTTTTTAAATCCTTGAGTAGAGCGGGGCACGTGAAATCCTGTTTGAAT  188910

  119     ATGGGGGGACCATCCTCCAAGCCTAAATACTCCTTAACGACCGATAGTGAACGAGTACCG  178
          |||||||||||||||||||||||||||||||||||||||||||||||||||| |||||||
  188911  ATGGGGGGACCATCCTCCAAGCCTAAATACTCCTTAACGACCGATAGTGAACAAGTACCG  188970

  179     TGAGGGAAAGGTGAAAAGAACCCCGGGAGGGGAGTGAAATAG-ATCCTGAAATCAAGTGC  237
          |||||||||||||||||||||||||||||||||||||||||| || ||||||||||||||
  188971  TGAGGGAAAGGTGAAAAGAACCCCGGGAGGGGAGTGAAATAGAAT-CTGAAATCAAGTGC  189029

  238     TTACAAACAGTTGGAGCTTTATATTTTTCTGCAAGAGCAATCTTGTATTGAATTTATATT  297
          |||||||||||||||||| ||||      |       |||  || ||||| |  ||| ||
  189030  TTACAAACAGTTGGAGCTCTATA------T-------CAA--TT-TATTG-A--TAT-TT  189069

  298     AGAGTGACAGCGTACCTTTTGCATAATGGGTCAGCGAGTTAATCTATGAAGCAAGCTTAA  357
          ||||||||||||||||||||||||||||||||||||||||||||||||||||||||||||
  189070  AGAGTGACAGCGTACCTTTTGCATAATGGGTCAGCGAGTTAATCTATGAAGCAAGCTTAA  189129

  358     GCCGTTAGGTGTAGGCATAGCGAAAGCAAGTCTTAATAGGGCG-TTTAGTTTTATGGATT  416
          |||||||||||||||| |||||||||||||||| ||||||||| ||||||||||||||||
  189130  GCCGTTAGGTGTAGGCGTAGCGAAAGCAAGTCTGAATAGGGCGTTTTAGTTTTATGGATT  189189

  417     AGACCCGAAACCAAGTGATCTAGTCATGACCAGATTGAAGGTGTGGTAAAACACACTGGA  476
          ||||||||||||||||||||||||||||||||||||||||||||||||||||||||||||
  189190  AGACCCGAAACCAAGTGATCTAGTCATGACCAGATTGAAGGTGTGGTAAAACACACTGGA  189249

 477      GGATCGAACCAGTTAATGTTGCAACATTATTGGATGAGTTGTGATTAGGGGTGAAAGGCC  536
          ||||||||||||||||||||||||||||||||||||||||||||||||||||||||||||
  189250  GGATCGAACCAGTTAATGTTGCAACATTATTGGATGAGTTGTGATTAGGGGTGAAAGGCC  189309

  537     AATCAAACTTGGAAATAGCTGGTTCTCCGCGAAATCTATTTAGGTAGAGCGTTGTATGTA  596
          ||||||||||||||||||||||||||||||||||||||||||||||||||||||||||||
```

```
189310  AATCAAACTTGGAAATAGCTGGTTCTCCGCGAAATCTATTTAGGTAGAGCGTTGTATGTA  189369

597     TGTTGTTGGGGGTAGAGCACTGGATAGACTAGGGGGATTCACCGTCTTACCAAATCTAAC  656
        ||||||||||||||||||||||||||||||||||||||||||||||||||||||||||||
189370  TGTTGTTGGGGGTAGAGCACTGGATAGACTAGGGGGATTCACCGTCTTACCAAATCTAAC  189429

657     CAAACTCCGAATACCAACAATTAATTATACAGCAGACACACTGCGGGTGCTAAGTCCGTG  716
        |||||||||||||||||||||||||||||||||| |||||| |||||||||||||||||
189430  TAAACTCCGAATACCAACAATTAATTATACAGCAGGCACACTACGGGTGCTAAGTCCGTG  189489

717     GTGAAAAGGGAAACAACCCAGATCACTATCTAAGGTCCCAAAATTACAGCTAAGTGGGGA  776
        ||||| ||||||||||||||||||||||||||||||||||||||||||||||||||||||
189490  GTGAAGAGGGAAACAACCCAGATCACTATCTAAGGTCCCAAAATTACAGCTAAGTGGGGA  189549

777     AGGAAGTAGAAAAACCATTACAGCTAGGAGGTTGGCTTGGAAGCAGCCATCCTTTAAAGA  836
        ||||||||||||||||||||||||||||||||||||||||||||||||||||||||||||
189550  AGGAAGTAGAAAAACCATTACAGCTAGGAGGTTGGCTTGGAAGCAGCCATCCTTTAAAGA  189609

837     AAGCGTAACAGCTCACTTGTCTAA-TAAGTTTTTCTGCGCTGAAAATGTACCGGGGCTAA  895
        |||||||||||||||||||||||| ||||||||||||||| |||||||||||||||||||
189610  AAGCGTAACAGCTCACTTGTCTAAATAAGTTTTTCTGCGCCGAAAATGTACCGGGGCTAA  189669

896     AGCTTGTATACCGAA  910
        |||| ||||||||||
189670  AGCT-GTATACCGAA  189683
```

Sequence 1 (reverse sequencing of the DNA fragment, Score = 1845 bits (999), Expect = 0.0 Identities = 1063/1093 (97%), Gaps = 10/1093 (1%)Strand=Plus/Minus

```
SA  1       CCGGATCACTATGACCGACTTTCGTCTCTGCTTGGCTTGTCAGCCTTGCAGTCAGGCAAG  60
            |||||||||||||||||||||||||||||||||||||||||||||| |||||||||||||
WRi 190805  CCGGATCACTATGACCGACTTTCGTCTCTGCTTGGCTTGTCAGCCTCGCAGTCAGGCAAG  190746

    61      CTTATGCCATTATACTATCAAGCTGATTTCCGACCAGCTCTAGCTTACCTTCGCACGCCT  120
            ||||||||||||||||||||||||||||||||||||||||||||||||||||||||||||
    190745  CTTATGCCATTATACTATCAAGCTGATTTCCGACCAGCTCTAGCTTACCTTCGCACGCCT  190686

    121     CCGTTACTTTTTAGGAGGCGACCGCCCCAGTCAAACTACCCACCATACAATGTCCTAGTT  180
            ||||||||||||||||||||||||||||||||||||||||||||||||||||||||||||
    190685  CCGTTACTTTTTAGGAGGCGACCGCCCCAGTCAAACTACCCACCATACAATGTCCTAGTT  190626

    181     CCAGATAATGAAACATAGTTAGATATTAAAAGTGTGAAGGGTGGTATCTCAATGAC-AGC  239
            |||||||||||||||||||||||||| |||||||| |||||||||||||||| |   | |
    190625  CCAGATAATGAAACATAGTTAGATATCAAAAGTGTAAAGGGTGGTATCTCAAGGTTGA-C  190567

    240     TCCATTATAGCTAGCGCCATAACTTCAAAG-CTTCCCACCTATCCTGCACATCACACTTT  298
```

```
              ||||||||||||||||||||||||||||| || ||||||||||||||||||||||||||
190566        TCCATTATAGCTAGCGCCATAACTTCAAAGTCT-CCCACCTATCCTGCACATCACACTTT   190508

299           TAACAGCAATGTAAAGCTATAGTAAAGGTGCACGGGGTCTCTTCGTCTAACCGCGGGTAC   358
              ||| ||||||||||||||||||||||||||||||||||||||||||||||||||||||||
190507        TAATAGCAATGTAAAGCTATAGTAAAGGTGCACGGGGTCTCTTCGTCTAACCGCGGGTAC   190448

359           CCCGCATCTGCACGGGGAATTCAATTTCGCTGAATTGATGTTGGAGACAGTGGAGAAATC   418
              |||||||||||||||||||||||||||||||||| |||||||||||||||||||||||||
190447        CCCGCATCTGCACGGGGAATTCAATTTCGCTGAAGTGATGTTGGAGACAGTGGAGAAATC   190388

419           GTTACGCCATTCGTGCGGGTCGGAACTTACCCGACAAGGAATTTCGCTACCTTAGGACCG   478
              ||||||||||||||||||||||||||||||||||||||||||||||||||||||||||||
190387        GTTACGCCATTCGTGCGGGTCGGAACTTACCCGACAAGGAATTTCGCTACCTTAGGACCG   190328

479           TCAGTGTTACGGCCGCCGTTTACTGGGGCTTCAATTCAGAGCTTGCACCCTTCCTATTAA   538
              ||||||||||||||||||||||||||||||||||||| ||||||||||||||||||||||
190327        TCAGTGTTACGGCCGCCGTTTACTGGGGCTTCAATTCGGAGCTTGCACCCTTCCTATTAA   190268

539           CCTTCCAGCACCGGGCAGGCGTCAGACCCTATACTTCCACTTACGTGTTTGCAGAGTCCT   598
              ||||||||||||||||||||||||||||||||||||||||||||||||||||||||||||
190267        CCTTCCAGCACCGGGCAGGCGTCAGACCCTATACTTCCACTTACGTGTTTGCAGAGTCCT   190208

599           GTGTTTTTAGTAAACAGTCGCTACTCCCTATTTTGTGCCACCTGC-CAATAGTTGCCTAA   657
              ||||||||||||||||||||||||||||||||||||||||||| | || |||||||||||
190207        GTGTTTTTAGTAAACAGTCGCTACTCCCTATTTTGTGCCACCTACTCA-TAGTTGCCTAA   190149

658           AAGCAGGTTACCCTTCTTCCGAAGTTACAGGTATAATTTGCCGAGTTCCTTCAACATCAT   717
              ||||||||||||||||||||||||||||||||||||||||||||||||||||||||||||
190148        AAGCAGGTTACCCTTCTTCCGAAGTTACAGGTATAATTTGCCGAGTTCCTTCAACATCAT   190089

718           TCTTTCAACACCTTAGTATACTCTACTCATCCACCAGTGTCGGTTTACGGTACGGCCTCA   777
              |||||||||||||||||||||||||||||||||||||||||||||| |||||||||||||
190088        TCTTTCAACACCTTAGTATACTCTACTCATCCACCAGTGTCGGTTTGCGGTACGGCCTCA   190029

778           TAAATATAAGTGCTATTTCCTGGAGCTTCTTTTAAGCATA-AGTCAATCCAATAAGACCT   836
              |||||||||||||||||||||||||||||||||||||||| | |||||||||||||| ||
190028        TAAATATAAGTGCTATTTCCTGGAGCTTCTTTTAAGCATAGA-TCAATCCAATAAGATCT   189970

837           ATACAAATACAAAACCCGTCACACTTAAGAGGTTTAGGAATATTAACCTAATTGCCATCG   896
              |||||||||| |||||||||||||||||||||||||||||||||||||||||||||||||
189969        ATACAAATACGAAACCCGTCACACTTAAGAGGTTTAGGAATATTAACCTAATTGCCATCG   189910

897           ACTACTCCTTTACGGACTCGCCTTANGAACCGACTAACCCTACGCAGATTAACTTAACGT   956
              ||||||||||||||||||||||||| ||||||||||||||||||||||||||||||||||
189909        ACTACTCCTTTACGGACTCGCCTTAGGAACCGACTAACCCTACGCAGATTAACTTAACGT   189850
```

```
957      AGGAAACCTTAGATTTTTGGTGAGAGTGTTTT-CACACTCTTTTACGCTACTTATGTCAG  1015
         ||||| |||||||||||||||||||||||||| |||||||||||||||||||||||||||
189849   AGGAACCCTTAGATTTTTGGTGAGAGTGTTTTTCACACTCTTTTACGCTACTTATGTCAG  189790

1016     CATTCTCACTTCCGATATCTCGAGTAGTCTTCTCAAACTAC-TTCACAGACTTACGGAAC  1074
         ||||||||||||||||||||| || ||| ||| ||||| || ||||||||||||||||||
189789   CATTCTCACTTCCGATATCTCCAGCAGTTTTCACAAACCACCTTCACAGACTTACGGAAC  189730

1075     GCTCCGCTACCGC  1087
         |||||||||||||
189729   GCTCCGCTACCGC  189717
```
